# Prompt-Guided Semantic Latent Direction Learning in Diffusion Models for Abstract Visual Concept Manipulation

**DOI:** 10.3390/jimaging12070279

**Published:** 2026-06-25

**Authors:** Mahzaib Khalid, Fangli Ying, Al-Garadi Ahmed Mohammed Atef, Aniwat Phaphuangwittayakul, Riyad Dhuny

**Affiliations:** 1 Department of Computer Science, East China University of Science and Technology, Shanghai 200237, China; yfangli@ecust.edu.cn (F.Y.); y30231667@mail.ecust.edu.cn (A.-G.A.M.A.); 2 International College of Digital Innovation, Chiang Mai University, Chiang Mai 50200, Thailand; aniwat.ph@cmu.ac.th; 3 Department of Creative Arts, Film and Media Technologies, University of Technology, Mauritius, Port Louis 11134, Mauritius; dhuny@utm.ac.mu

**Keywords:** diffusion models, stable diffusion, concept-vector learning, prompt-guided learning, semantic manipulation, image-to-image editing, bottleneck feature injection, abstract visual concepts

## Abstract

Diffusion-based generative models achieve high-fidelity image synthesis; however, controlling internal representations for abstract visual concepts remains challenging due to the ambiguity of textual descriptions. In this work, we propose a prompt-guided concept-vector learning framework for the controllable manipulation of such concepts without requiring external human-annotated image pairs, segmentation masks, identity labels, or manually annotated editing targets. The method introduces a learnable concept vector optimized in the bottleneck (mid-block) feature space of a pretrained Stable Diffusion U-Net, while keeping all pretrained model parameters frozen. A multi-prompt data generation strategy based on paired positive and neutral prompts provides weak semantic guidance for capturing the target concept direction and reducing dependence on a single prompt formulation. The learned vector is further applied in an image-to-image setting through controlled noise injection and concept-guided denoising, enabling the semantic modification of real images while preserving structural content. The concept strength is controlled by a scaling parameter γ, while the image-to-image noise strength is controlled by β, allowing for a practical balance between semantic modification and structural fidelity. Experiments are conducted on two main abstract concepts, *perfect skin* and *peaceful lake*, with additional qualitative analysis on subjective portrait-level concepts. Quantitative evaluation using SSIM, LPIPS, and CLIP similarity demonstrates that the proposed method improves semantic alignment while maintaining structural preservation compared with Stable Diffusion image-to-image baselines. A human preference study further shows that concept-injected outputs are preferred in 76.0% of responses for *perfect skin* and 85.7% for *peaceful lake*. Ablation studies further demonstrate the controllability and robustness of the proposed framework. Overall, the method provides a simple and parameter-efficient approach for interpretable concept-level manipulation in diffusion models.

## 1. Introduction

Diffusion-based generative models have become a dominant paradigm for high-fidelity image synthesis, achieving strong performance across a wide range of visual generation tasks [1,2]. In particular, latent diffusion frameworks such as Stable Diffusion enable efficient image generation in compressed latent spaces while maintaining high perceptual quality [3]. Despite these advances, controlling the internal representations of such models remains challenging, especially for abstract visual concepts that lack precise textual definitions. Text-to-image diffusion models rely heavily on linguistic conditioning, assuming that semantic intent can be adequately expressed through text prompts. While this assumption is effective for well-defined attributes such as object categories, colors, or simple style cues, it becomes limiting for abstract and subjective concepts, such as facial aesthetics, emotional portrait tone, or scene atmosphere. These concepts are continuous in their visual representation and difficult to describe explicitly, often leading to inconsistent or weak control when manipulated through prompts alone.

Existing techniques for controllable generation, including classifier-free guidance, prompt-based editing, and image-to-image diffusion, further emphasize this reliance on textual descriptions or externally defined conditions [4,5,6]. Although such methods provide practical control for many editing tasks, they often struggle when the desired concept is subjective, implicit, or difficult to specify using a single prompt. Several recent works have explored latent space manipulation in diffusion models to provide more direct semantic control. These approaches often rely on labeled data, predefined attributes, auxiliary models, or vision-language embeddings [7,8]. While effective in specific settings, they may be limited when the target concept lacks clear annotations or well-defined linguistic boundaries. Unsupervised or weakly guided approaches have also been proposed to identify latent directions within diffusion models [9,10,11], but the resulting directions can be difficult to associate with a specific user-defined concept or may behave inconsistently across different inputs.

In this work, we propose a prompt-guided concept-vector learning framework for the controlled manipulation of abstract visual concepts. The approach introduces a learnable concept vector optimized within the bottleneck (mid-block) feature representation of a pretrained diffusion U-Net, while keeping all pretrained model parameters frozen. By operating at this intermediate representation, the method targets a region of the network where high-level semantic features are aggregated, enabling global semantic modifications through a simple additive transformation. To construct the learning signal, we employ a positive–neutral prompt pairing strategy in which images are generated using concept-enhanced positive prompts, while conditioning is performed using corresponding neutral prompts. The positive and neutral prompt pools provide weak semantic guidance for the intended concept direction, but no external human-annotated image pairs, pixel-level labels, segmentation masks, identity labels, or manually annotated editing targets are required. Therefore, the proposed framework should be understood as prompt-guided concept-vector learning with self-supervised noise-prediction optimization, rather than fully unsupervised semantic discovery.

A multi-prompt data generation strategy is further used to capture variability in linguistic descriptions, reducing dependence on a single prompt formulation and improving concept coverage across different visual contexts. The learned concept vector is then applied in an image-to-image setting by incorporating controlled noise injection and bottleneck-level concept injection during denoising [6,12]. This enables the semantic modification of real images while preserving structural content. A scaling parameter γ controls the strength of the learned concept, while the image-to-image noise strength β controls the trade-off between structural fidelity and editing freedom.

The main contributions of this work are summarized as follows:

- We propose a prompt-guided concept-vector learning framework for the controllable manipulation of abstract visual concepts without requiring external human-annotated image pairs, pixel-level labels, segmentation masks, identity labels, or manually annotated editing targets.
- We introduce a positive–neutral multi-prompt pairing strategy and a parameter-efficient bottleneck injection mechanism, where only a compact concept vector is learned while the pretrained VAE, text encoder, and U-Net remain frozen.
- We extend the learned concept vector to real-image editing through an image-to-image pipeline, enabling controllable semantic manipulation through γ and β, and validate the framework through quantitative, qualitative, human preference, baseline comparison, and ablation analyses.

## 2. Related Work

### 2.1. Controllable Generation in Diffusion Models

Diffusion models have significantly advanced high-fidelity image synthesis and controllable text-to-image generation [1,2]. A common strategy for improving controllability is to guide the denoising process using conditioning signals. Classifier-guided and classifier-free guidance methods steer generation toward desired attributes, but they depend on predefined conditioning information and assume that the target semantics can be sufficiently expressed through such signals [4]. Vision-language models further support semantic alignment between text and images, enabling natural-language-based control [7]. However, text-based control remains limited when the target concept is abstract, subjective, or difficult to describe precisely.

Several editing methods improve controllability by manipulating internal attention maps or incorporating additional conditions. Prompt-based editing approaches, such as cross-attention control, modify attention maps to enable prompt-level or localized semantic changes [13]. These methods are effective for many object-level or attribute-level edits, but they remain sensitive to prompt formulation and token alignment. Other methods introduce structural guidance signals, such as edges, depth, pose, or adapter-based conditions, to improve spatial control [14,15]. For example, ControlNet injects external structural conditions into pretrained diffusion models while preserving the original model weights [14]. Although these approaches provide strong control for structured editing tasks, they rely on externally defined conditions and are not specifically designed to learn compact representations of abstract semantic attributes.

Image-to-image diffusion frameworks provide another practical direction for real-image editing by applying partial noise injection followed by conditional denoising [6,12]. These methods can preserve the input structure while enabling semantic transformation, making them useful for real-image manipulation. Nevertheless, their editing behavior is still largely governed by text prompts or external guidance, which can be insufficient for concepts such as facial aesthetics, emotional portrait tone, or scene atmosphere. In contrast, our work aims to learn an explicit concept vector inside the diffusion model, enabling the controllable manipulation of abstract visual concepts beyond prompt-only conditioning.

### 2.2. Latent Space Interpretation and Semantic Vector Learning

Recent studies have explored the internal representation space of diffusion models to understand and manipulate semantic attributes more directly [11,16]. Prior work suggests that diffusion models encode meaningful semantic structure in latent or intermediate feature spaces, allowing visual attributes to be modified through directional operations [11]. Related studies have also explored diffusion autoencoder representations and rare-concept generation with pretrained diffusion models, further indicating that diffusion models contain useful internal representations for semantic control [17,18]. This has motivated methods for discovering or learning semantic directions that correspond to interpretable visual changes [9,19].

Existing latent-direction methods differ in the amount of supervision they require. Some approaches use external signals, such as attribute classifiers, predefined labels, or vision-language embeddings, to identify controllable directions [7]. These methods can provide useful semantic guidance, but they are often limited to predefined categories and may require additional models or annotations. Other approaches attempt to discover latent directions without manual labels [10]. While such methods reduce annotation cost, the discovered directions may lack clear semantic grounding, making it difficult to associate them with a specific user-defined concept or to apply them consistently across different inputs.

Recent work has also explored weakly supervised or self-guided strategies for improving the semantic alignment of latent directions [20,21]. These methods demonstrate that meaningful transformations can be obtained with reduced dependence on manually labeled image data. However, they often focus on well-defined attributes or rely on assumptions about the organization of the latent space. As a result, capturing abstract and subjective concepts, such as skin quality, calmness, dramatic mood, or melancholic atmosphere, remains challenging. Another limitation is that many existing methods do not explicitly model the variability of a concept across different linguistic expressions and visual contexts, which can make the learned direction sensitive to specific prompts or input images [22].

In contrast, the proposed framework focuses on prompt-guided concept-vector learning for abstract and linguistically ambiguous concepts. Positive and neutral prompt pools provide weak semantic guidance, while the method does not require external human-annotated image pairs, segmentation masks, identity labels, or manually annotated editing targets. By using paired prompts and multi-prompt synthetic data generation, the method captures multiple visual expressions of the same concept and reduces dependence on a single prompt formulation. The learned concept vector is then injected into the U-Net bottleneck representation and applied in an image-to-image pipeline, enabling the controllable semantic manipulation of real images while preserving structural content. This design provides a simple and parameter-efficient alternative to prompt-only editing, token-based personalization, and broader latent-direction discovery methods.

## 3. Materials and Methods

### 3.1. Overview of the Framework

**Figure 1:**
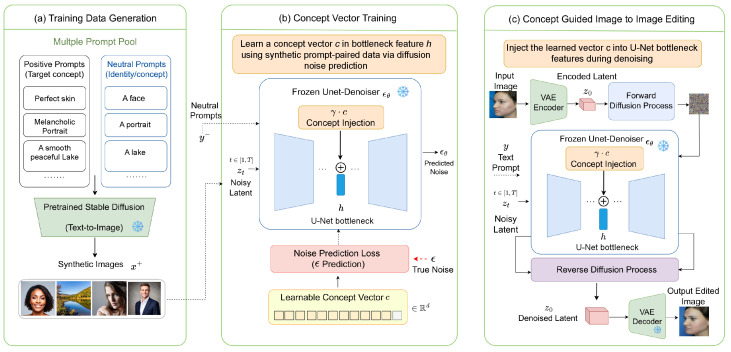
Overview of the proposed framework. (**a**) Multi-prompt data generation constructs a diverse synthetic dataset using positive prompts that describe the target concept and neutral prompts that describe the corresponding baseline semantics through a pretrained Stable Diffusion text-to-image model. (**b**) Concept-vector optimization learns a semantic concept vector c in the U-Net bottleneck feature representation *h* by minimizing the diffusion noise-prediction loss, while keeping all pretrained model parameters frozen. The positive and neutral prompt pools provide weak semantic guidance for the target concept direction. (**c**) Image-to-image editing injects the learned vector c, scaled by the concept strength γ, into the U-Net bottleneck feature representation during denoising, enabling controllable semantic manipulation of real images while preserving structural fidelity. Arrows indicate the processing flow from prompt-based data generation to concept-vector optimization and real-image editing.

The proposed framework addresses the challenge of controlling abstract and subjective visual concepts in diffusion models, which are difficult to reliably express through text prompts alone. The central idea is to enable the direct manipulation of semantic attributes by learning a concept vector c in the U-Net bottleneck feature representation *h* of a pretrained diffusion model. The framework follows a prompt-guided concept-vector learning strategy and consists of three main stages, as illustrated in Figure 1. First, a multi-prompt data generation strategy is employed to construct a diverse synthetic dataset that captures the variability of a target concept. Specifically, a prompt pool containing positive prompts, which describe the desired concept, and neutral prompts, which capture baseline semantics, is used with a pretrained Stable Diffusion model to generate training samples. Second, the synthetic dataset is used to learn a concept vector $c\in R^{d}$ while keeping all pretrained model parameters fixed. The vector is injected into the bottleneck (mid-block) representation of the U-Net during the denoising process. The training objective follows the standard diffusion noise-prediction loss, allowing the concept vector to capture the semantic shift between neutral and concept-enhanced representations without requiring external human-annotated image pairs, segmentation masks, identity labels, or manually annotated editing targets. Finally, the learned concept vector is applied within an image-to-image generation setting, where it modifies real images through bottleneck feature manipulation during denoising. By injecting a scaled version of the vector, γc, into the U-Net bottleneck representation, the framework enables controllable semantic editing while preserving the structural content of the input image. Overall, the method provides a parameter-efficient mechanism for guiding the denoising process through a learned bottleneck-level perturbation, enabling the consistent manipulation of abstract visual concepts across synthetic and real-image domains.

### 3.2. Multi-Prompt Synthetic Dataset Generation

To effectively model abstract and subjective visual concepts, the proposed framework employs a multi-prompt data generation strategy to construct a diverse and semantically rich synthetic dataset. A single-prompt setting may capture only a narrow representation of a target concept, especially when the concept is subjective or visually diverse. To reduce this dependence on one textual formulation, the proposed method uses a prompt pool consisting of multiple textual descriptions that represent different aspects of the target concept. Specifically, the prompt pool is divided into two categories: (i) positive prompts, which explicitly describe the desired semantic attribute, and (ii) neutral prompts, which provide baseline descriptions without emphasizing the target concept. This positive–neutral prompt design enables the framework to model the semantic shift between concept-specific and general visual representations. Representative examples of the prompt pool for the evaluated concepts are shown in Table 1.

Each prompt in the pool is sampled to generate images using a pretrained Stable Diffusion text-to-image model, resulting in a synthetic dataset that captures both intra-concept variability and contextual diversity. During training, images generated from positive prompts are paired with corresponding neutral prompts. The positive and neutral prompts provide weak semantic guidance by defining the desired concept shift, but the training process does not require real paired images, segmentation masks, identity labels, or human-annotated edit targets. The learning signal is obtained from synthetic prompt-paired samples and the diffusion noise-prediction objective, while all pretrained diffusion model parameters remain frozen. In this sense, the learning process is prompt-guided with respect to the target concept and self-supervised with respect to image annotations, rather than fully unsupervised.

The use of multiple semantically related prompts allows the generated samples to reflect different visual manifestations of the same concept, reducing dependence on any single linguistic formulation. For instance, variations in prompt phrasing can lead to differences in texture, illumination, identity, and composition while preserving the underlying semantic attribute. Randomized prompt sampling is applied during dataset generation to further improve diversity and encourage the learned vector to represent the shared concept direction rather than a prompt-specific artifact. As a result, the constructed dataset maintains a balance between semantic consistency and visual variation, providing a robust foundation for subsequent concept-vector optimization. The effectiveness of this multi-prompt design is further examined in the single-prompt versus multi-prompt ablation study.

### 3.3. Prompt-Guided Concept Vector Optimization

Building on the multi-prompt synthetic dataset, the proposed framework learns a semantic concept vector through prompt-guided optimization in the U-Net bottleneck feature space of a pretrained diffusion model. Specifically, the U-Net denoiser is kept frozen, and only a learnable vector $c\in R^{d}$ is optimized to capture the semantic variation associated with a target concept. Let $z_{t}$ denote a noisy latent sample at diffusion timestep *t*, and let $y^{-}$ represent the corresponding neutral prompt. The pretrained U-Net denoiser is denoted by $\epsilon_{\theta }\left(\cdot \right)$, which predicts the noise component at each timestep. During the forward pass, the intermediate feature representation at the U-Net bottleneck (mid-block) is denoted by $h_{t}$. To incorporate semantic information, the concept vector c is injected into this representation, resulting in the following modified feature:

(1) $$h_{t}^{\prime }=h_{t}+\gamma c,$$

where γ is a scalar controlling the strength of concept injection. In the main experiments, γ is fixed during the reverse denoising process and selected at inference time to control the edit intensity. The same learned vector c is shared across all images of a given concept. Setting γ=0 reduces the model to the image-to-image baseline without concept injection, while increasing γ strengthens the learned semantic direction. Excessively large values of γ may cause over-editing or artifacts, especially when combined with high noise levels.

The training objective follows the standard diffusion noise-prediction formulation. Given a training image x∼D, a timestep t∼U(1,T), and Gaussian noise ϵ∼N(0,I), the concept vector is optimized as

(2) $$c^{*}=\arg\min_{c}E_{x∼D,\;t∼U(1,T),\;\epsilon ∼N(0,I)}{∥\epsilon -{\hat{\epsilon }}_{\theta }\left(z_{t},\pi \left(y^{-}\right),t;c,\gamma \right)∥}_{2}^{2},$$

where ϵ denotes the ground-truth Gaussian noise, $\pi (y^{-})$ represents the embedding of the neutral prompt, and ${\hat{\epsilon }}_{\theta }\left(\cdot ;c,\gamma \right)$ denotes the noise prediction obtained when the bottleneck feature is modified using $h_{t}^{\prime }=h_{t}+\gamma c$. During optimization, all pretrained parameters of the VAE, text encoder, and U-Net remain frozen, and only c is updated. This objective allows the concept vector c to capture the semantic shift between neutral and concept-enhanced representations by modifying the internal features of the U-Net without changing the pretrained diffusion model. Since the supervision signal is obtained from synthetic prompt-paired samples and the diffusion noise-prediction objective, the learning process is self-supervised with respect to image annotations while being weakly guided by the positive–neutral prompt design. By optimizing only c, the framework learns a compact transformation associated with the target concept. The learned concept vector can then be applied across different inputs to produce consistent semantic modifications, enabling the controllable editing of abstract attributes such as enhanced facial appearance or modified scene atmosphere while preserving the structural content of the original image.

### 3.4. Rationale for Bottleneck-Level Concept Injection

The concept vector is injected into the U-Net bottleneck because this representation provides a compact and high-level feature space for influencing the global denoising trajectory. In the U-Net architecture, encoder features are more closely related to local spatial details such as edges, textures, and low-level structures, while decoder features contribute to the reconstruction and refinement of image details. In contrast, the bottleneck aggregates information from the encoder at a lower spatial resolution and is therefore suitable for representing global semantic attributes. Since the target concepts considered in this work, such as skin smoothness, lake atmosphere, emotional portrait tone, and dramatic lighting, correspond to broad appearance-level or scene-level changes, bottleneck-level injection provides an effective and parameter-efficient control point. We do not inject the learned vector into cross-attention layers because cross-attention primarily controls the interaction between text tokens and image features. Manipulating cross-attention can be effective for token-specific or localized prompt editing, but it often requires explicit token alignment, attention-map control, or layer-wise intervention. In contrast, our objective is to learn a reusable concept vector from paired positive and neutral prompts without modifying the text encoder, attention maps, or pretrained U-Net parameters. Bottleneck injection therefore provides a simpler mechanism for learning and applying a global concept direction. A limitation of this design is that the learned vector acts as a global semantic perturbation rather than a spatially localized control signal. Therefore, the proposed method is not designed for precise region-specific editing, such as modifying only a small facial region while leaving the rest of the image unchanged. Such tasks would require additional spatial masks, attention control, or region-specific conditioning. The proposed framework is better suited to global appearance, style, and atmosphere manipulation, where the target concept can be expressed as a broad shift in the denoising representation.

### 3.5. Image-to-Image Editing Pipeline

To extend the learned concept representation to real-image editing, we introduce an image-to-image (I2I) pipeline that integrates the learned concept vector into the diffusion denoising process. This pipeline enables controllable manipulation of abstract semantic attributes by combining partial noise-based initialization with bottleneck-level concept injection. An input image $x_{0}$ is first encoded into the latent space using the pretrained VAE encoder, producing a latent representation $z_{0}$. This latent code is then perturbed with Gaussian noise according to the diffusion forward process:

(3) $$z_{t}=\sqrt{{\bar{\alpha }}_{t}}z_{0}+\sqrt{1-{\bar{\alpha }}_{t}}\;\epsilon ,$$

where ${\bar{\alpha }}_{t}=\prod_{s=1}^{t}\alpha_{s}$ denotes the cumulative noise schedule and ϵ∼N(0,I) is Gaussian noise. In the image-to-image setting, the noise strength parameter β∈[0,1] determines the starting denoising level. Lower values of β preserve more of the original image structure, while higher values allow stronger semantic transformation.

During the reverse denoising process, the learned concept vector c is injected into the U-Net bottleneck representation at each timestep:

(4) $$h_{t}^{\prime }=h_{t}+\gamma c,$$

where γ controls the strength of the semantic modification. This additive perturbation is applied within the mid-block of the U-Net and influences the denoising trajectory without modifying the pretrained network parameters. The denoising process remains conditioned on the prompt embedding π(y), while the concept vector directly modifies the intermediate bottleneck feature representation. The resulting noise prediction can be written as

(5) $${\hat{\epsilon }}_{\theta }\left(z_{t},\pi \left(y\right),t;c,\gamma \right),$$

where the effect of the concept vector is incorporated through the modified bottleneck feature $h_{t}^{\prime }$ during the U-Net forward pass.

By combining prompt conditioning with bottleneck-level feature modification, the framework enables the controlled manipulation of abstract semantic attributes in real images. The proposed I2I pipeline provides a practical mechanism for applying the learned concept representation to image editing, enabling the continuous control of semantic strength through γ while balancing structural fidelity through β.

### 3.6. Experimental Setup

We evaluate the proposed framework using two main concepts, *perfect skin* and *peaceful lake*, for full quantitative and qualitative analysis. These concepts represent appearance-level facial editing and scene-atmosphere editing, respectively. To further examine concept diversity, we additionally include two subjective portrait-level concepts, *melancholic portrait* and *dramatic portrait*, in the qualitative analysis. All experiments are conducted using Stable Diffusion 1.5 as the pretrained diffusion backbone. For each concept, we construct a synthetic training dataset using the multi-prompt strategy described above. Positive prompts are used to generate concept-enhanced images, while corresponding neutral prompts are used as conditioning inputs during concept-vector optimization. The positive and neutral prompt pools provide weak semantic guidance for the intended concept direction, but no external human-annotated image pairs, segmentation masks, identity labels, or manually annotated editing targets are used. For each concept, 500 synthetic images are generated at a resolution of 512×512.

This dataset size is chosen because the proposed method optimizes only a compact 1280-dimensional concept vector while keeping the pretrained VAE, text encoder, and U-Net frozen. As a result, large-scale concept-specific training data is not required. In our experiments, 500 synthetic samples provide sufficient prompt and appearance diversity while keeping data generation and optimization cost manageable. The concept vector is trained using the standard diffusion noise-prediction objective with the Adam optimizer and a learning rate of $1\times 10^{-4}$ for 5000 optimization steps. This setting provides a practical balance between stable concept-vector learning and computational efficiency.

For real-image editing, we use the image-to-image pipeline described in Section 3.5. The noise strength β controls how strongly the input image latent is perturbed before denoising, while the concept strength γ controls the magnitude of the learned concept vector injected into the U-Net bottleneck. Lower β values preserve more input structure but limit semantic editability, whereas higher β values enable stronger transformations but increase the risk of identity or structural drift. Similarly, larger γ values strengthen the target concept but may cause over-editing when set too high. Therefore, moderate settings are used for the main real-image experiments, and additional ablation analyses examine more extreme β–γ combinations.

For real-image evaluation, we use 67 images for the *perfect skin* concept and 55 images for the *peaceful lake* concept, after manual collection and quality screening to retain only clear and relevant samples for each editing scenario. All quantitative metrics are computed using a clean raw-image protocol, where the original input image, the no-concept output, and the concept-injected output are saved as separate images. This avoids the influence of labels, figure margins, or combined visualization layouts on the evaluation. The assessment focuses on whether the method can preserve the input structure while producing semantic changes aligned with the target concept.

### 3.7. Evaluation Metrics

We use three complementary metrics to evaluate image editing quality: SSIM, LPIPS, and CLIP similarity. SSIM and LPIPS measure preservation of the input image, while CLIP similarity measures semantic alignment with the target concept. Since the evaluated concepts are abstract and partly subjective, these metrics are interpreted together with qualitative results and the human preference study.

**SSIM:** The Structural Similarity Index Measure [23] evaluates structural consistency between the input image and the edited output. A higher SSIM value indicates better preservation of layout and spatial structure. However, a very high SSIM may also indicate conservative editing if the target concept is weakly expressed.

**LPIPS:** Learned Perceptual Image Patch Similarity [24] measures perceptual distance using deep visual features. In this work, LPIPS is computed between the input image and the edited output. A lower LPIPS value indicates that the edited image remains perceptually closer to the input. However, stronger semantic editing can increase LPIPS because the output intentionally changes the original appearance.

**CLIP Similarity:** CLIP similarity [7] measures semantic alignment between the edited image and the target concept prompt. It is computed as the cosine similarity between the CLIP image embedding of the edited output and the CLIP text embedding of the target concept. A higher CLIP value indicates stronger semantic agreement with the intended concept. However, CLIP is only an approximate proxy for subjective attributes such as peacefulness, dramatic mood, or perceived skin quality.

Overall, a successful edit should not be judged by a single metric alone. High preservation scores may indicate limited semantic change, while strong semantic editing may slightly reduce structural or perceptual similarity. Therefore, we report aggregate scores over the full real-image test sets and interpret SSIM, LPIPS, and CLIP jointly with qualitative examples and human preference results.

## 4. Results

### 4.1. Quantitative Results

To quantitatively evaluate the proposed framework, we compare our method with a standard Stable Diffusion image-to-image baseline and an ablated variant without concept-vector injection. The evaluation is conducted on 67 real images for the “perfect skin” concept and 55 real images for the “peaceful lake” concept. SSIM and LPIPS are computed between the edited image and the corresponding input image, while CLIP similarity is computed between the edited image and the target concept prompt.

Table 2 presents the quantitative results for the “perfect skin” concept. Compared with Stable Diffusion, the proposed method with concept injection improves SSIM from 0.6636 to 0.7023 and CLIP similarity from 0.2460 to 0.2491, while reducing LPIPS from 0.2478 to 0.2370. Compared with the no-concept ablation, concept injection slightly improves SSIM and CLIP similarity, while LPIPS increases slightly from 0.2314 to 0.2370. This indicates that the learned concept vector improves semantic alignment while maintaining competitive structural and perceptual consistency.

Table 3 summarizes the quantitative results for the “peaceful lake” concept. The proposed method with concept injection achieves the highest SSIM score, improving from 0.4578 for Stable Diffusion to 0.4854. It also obtains the highest CLIP similarity, improving from 0.2663 for Stable Diffusion and 0.2820 for the no-concept ablation to 0.2871. Stable Diffusion obtains the lowest LPIPS value, indicating stronger perceptual closeness to the input image; however, this may also reflect a more conservative transformation. Within our framework, concept injection improves SSIM, LPIPS, and CLIP compared with the no-concept ablation, demonstrating the contribution of the learned concept vector.

Overall, the quantitative results show that the proposed method improves SSIM and CLIP similarity for both evaluated concepts compared with the Stable Diffusion baseline. For the “perfect skin” concept, the method also reduces LPIPS compared with Stable Diffusion, indicating improved perceptual consistency. For the “peaceful lake” concept, Stable Diffusion achieves lower LPIPS, but the proposed method provides stronger semantic alignment and better structure preservation according to CLIP and SSIM. These results support the effectiveness of the learned concept vector, while also showing the need to interpret preservation and semantic-alignment metrics jointly for subjective visual concepts.

### 4.2. Human Preference Study

Since the proposed framework targets abstract and subjective visual concepts, automatic metrics alone cannot fully capture perceived concept quality. We therefore conducted a human preference study to evaluate whether the concept-injected outputs better represent the intended concepts. Participants were shown anonymized comparisons consisting of the original image and two edited outputs. One edited output was generated without concept-vector injection, while the other was generated with the learned concept vector. The order of the two edited outputs was randomized to reduce ordering bias. Participants were asked to select which output better represented the target concept while preserving the original image structure. The available choices were the concept-injected output, the no-concept output, and “no clear preference”.

**Figure 2:**
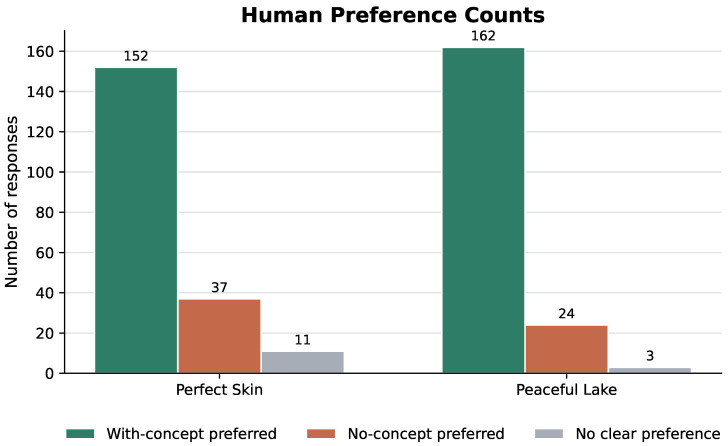
Human preference study for subjective concept editing. Participants compared no-concept and concept-injected outputs while viewing the original image and selected which output better represented the target concept while preserving image structure. For the “perfect skin” concept, the concept-injected output was preferred in 152 out of 200 responses (76.0%). For the “peaceful lake” concept, the concept-injected output was preferred in 162 out of 189 responses (85.7%). These results indicate that human perception is strongly aligned with the intended concept direction.

As shown in Figure 2, the concept-injected outputs were preferred by most participants for both evaluated concepts. For the “perfect skin” concept, the concept-injected output was preferred in 152 out of 200 responses, corresponding to 76.0%. For the “peaceful lake” concept, the concept-injected output was preferred in 162 out of 189 responses, corresponding to 85.7%. In contrast, the no-concept outputs were preferred in only 18.5% and 12.7% of responses for the two concepts, respectively. These results indicate that human judgments are strongly aligned with the intended semantic direction of the learned concept vectors. They also complement the automatic metrics, especially for subjective concepts where CLIP similarity may not fully capture perceived skin quality, calmness, or peaceful atmosphere.

### 4.3. Qualitative Results

**Figure 3:**
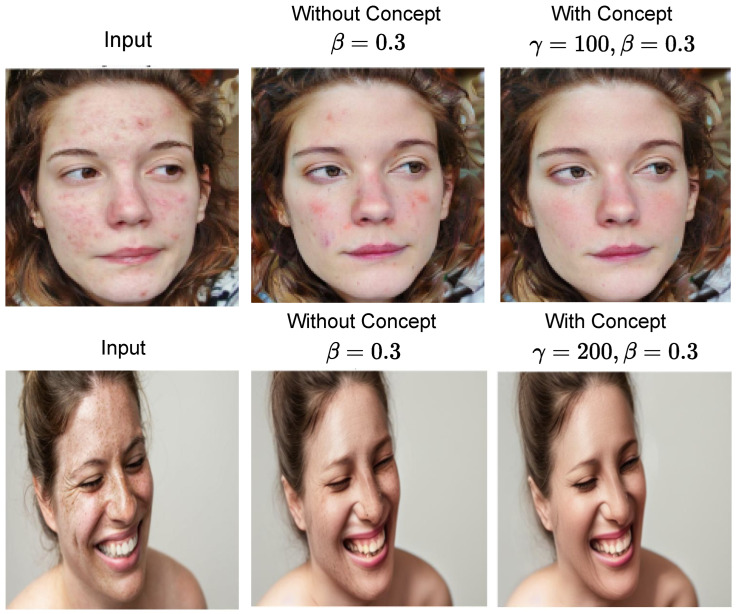
Qualitative results for the “perfect skin” concept. Each row shows, from left to right: the original image, the output without concept injection, and the output with the learned concept vector applied. All results are generated with a fixed noise level (β=0.3), while the concept strength γ is varied across rows. Concept injection smooths skin texture and reduces visible imperfections while preserving facial identity and structural consistency.

The qualitative results further illustrate how the learned concept vectors modify abstract visual attributes while preserving the main structure of the input image. Figure 3 shows real-image editing results for the “perfect skin” concept. Each row compares the original image, the output without concept injection, and the output with the learned concept vector applied. The no-concept outputs preserve the general facial structure but retain visible skin imperfections. In contrast, the concept-injected outputs show smoother skin texture and reduced blemishes while maintaining facial identity and overall structure. Increasing γ strengthens the concept effect, demonstrating controllable semantic editing through the learned concept vector.

**Figure 4:**
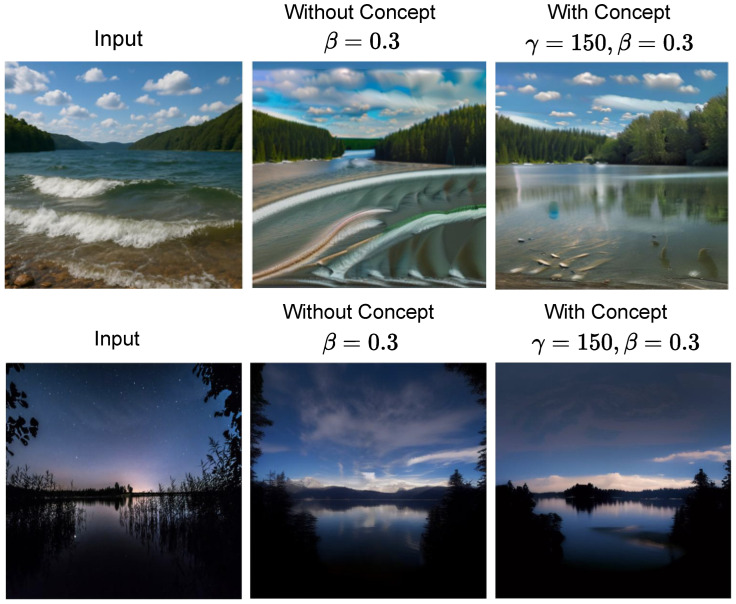
Qualitative results for the “peaceful lake” concept. Each row shows, from left to right: the original image, the output without concept injection, and the output with the learned concept vector applied. All results are generated with a fixed noise level (β=0.3) and concept strength (γ=150). Concept injection produces smoother water surfaces, more coherent reflections, and a more tranquil visual appearance while preserving overall scene structure.

Figure 4 presents qualitative results for the “peaceful lake” concept. Each row compares the original image, the output without concept injection, and the output with the learned concept vector applied. Without concept injection, the generated outputs may contain less coherent water surfaces or reflections. With concept injection, the outputs show smoother water textures, more coherent reflections, and a calmer visual atmosphere, aligned with the target concept. These results indicate that the learned concept vector can also manipulate scene-level atmospheric attributes while preserving the overall scene structure.

**Figure 5:**
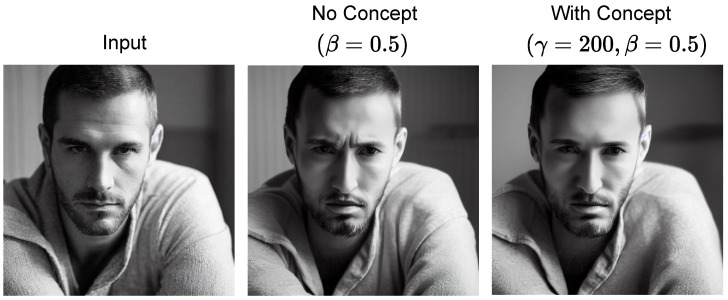
Qualitative result for the additional subjective concept “melancholic portrait”. Compared with the input and no-concept output, concept injection produces a more subdued and emotionally restrained portrait atmosphere under β=0.5 and γ=200. This example demonstrates applicability to subjective emotional concepts, although such effects remain sensitive to the concept strength and noise level.

To examine concept diversity beyond the two main evaluation settings, we additionally test subjective portrait-level concepts. Figure 5 shows the result for the “melancholic portrait” concept. Compared with the input and no-concept output, concept injection preserves the portrait structure while producing a more subdued and emotionally restrained appearance. This example suggests that the proposed framework can capture subjective emotional attributes in addition to texture-level and scene-level concepts.

**Figure 6:**
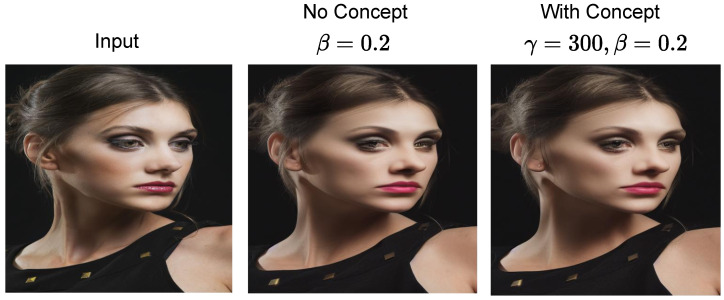
Qualitative result for the additional subjective concept “dramatic portrait”. Compared with the input and no-concept output, concept injection enhances contrast, lighting intensity, and cinematic portrait mood under β=0.2 and γ=300, while preserving the overall facial structure. This example further demonstrates the ability of the proposed framework to manipulate subjective portrait-level concepts beyond the main texture-level and scene-level settings.

Figure 6 further evaluates another subjective portrait-level concept, “dramatic portrait”. Compared with the input and no-concept output, concept injection enhances contrast, lighting intensity, and cinematic portrait mood while preserving the overall facial structure. Together with the melancholic portrait example, this result shows that the proposed framework can extend beyond the two main concepts and manipulate additional subjective portrait-level attributes. However, such effects remain dependent on the selected concept strength and noise level, indicating the need for careful control of γ and β.

### 4.4. Comparison with Existing Image Editing Methods

To better position the proposed framework, we compare it with representative diffusion-based image editing and concept-manipulation methods. Table 4 summarizes the differences in supervision requirements, trainable parameters, strengths, and limitations. The comparison includes prompt-based image-to-image editing, attention-based prompt editing, instruction-guided editing, token-based concept learning, self-discovered latent directions, and the proposed bottleneck-level concept-vector learning strategy.

**Figure 7:**
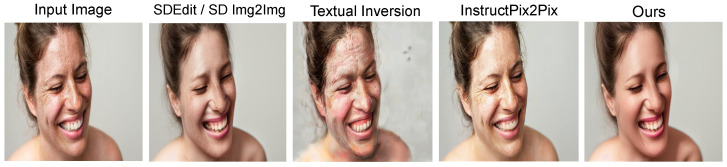
Qualitative comparison with representative image-editing baselines for the “perfect skin” concept. SDEdit/Stable Diffusion Img2Img preserves the input structure but provides only implicit prompt-based control. Textual Inversion introduces visible artifacts and unstable identity preservation, while InstructPix2Pix produces a relatively mild concept change. The proposed method provides explicit concept-strength control through γ and achieves a more stable global enhancement of the learned concept.

Prompt-to-Prompt is included as a method-level comparison because it primarily relies on cross-attention token alignment for prompt editing, whereas the present task focuses on real-image abstract concept manipulation through a learned bottleneck concept vector. Compared with prompt-only and instruction-based editing methods, the proposed method learns an explicit concept vector that can be reused across real images and controlled continuously through γ. Unlike full model fine-tuning, all pretrained diffusion components remain frozen, and only a 1280-dimensional vector is optimized for each concept. The method is therefore lightweight, but it is mainly suited to global semantic, appearance-level, or atmosphere-level edits rather than precise localized or geometry-aware manipulation. We further provide a qualitative comparison on the “perfect skin” concept using SDEdit/Stable Diffusion Img2Img [3,6], Textual Inversion [25], and InstructPix2Pix [12]. As shown in Figure 7, SDEdit/Stable Diffusion Img2Img preserves the main facial structure but provides only implicit prompt-based control. Textual Inversion produces stronger appearance changes but introduces visible artifacts and unstable identity preservation. InstructPix2Pix preserves the facial structure reasonably well, but the concept enhancement remains mild. In contrast, the proposed method provides explicit concept-strength control through γ and produces a more stable global enhancement of the target concept while preserving the main facial structure.

### 4.5. Ablation and Robustness Analysis

#### 4.5.1. Robustness Under Coupled β–γ Settings

We analyze the coupled effect of the image-to-image noise strength β and the concept injection strength γ. The parameter β controls how strongly the input latent is perturbed before reverse denoising, while γ controls the magnitude of the learned concept vector injected into the U-Net bottleneck.

**Figure 8:**
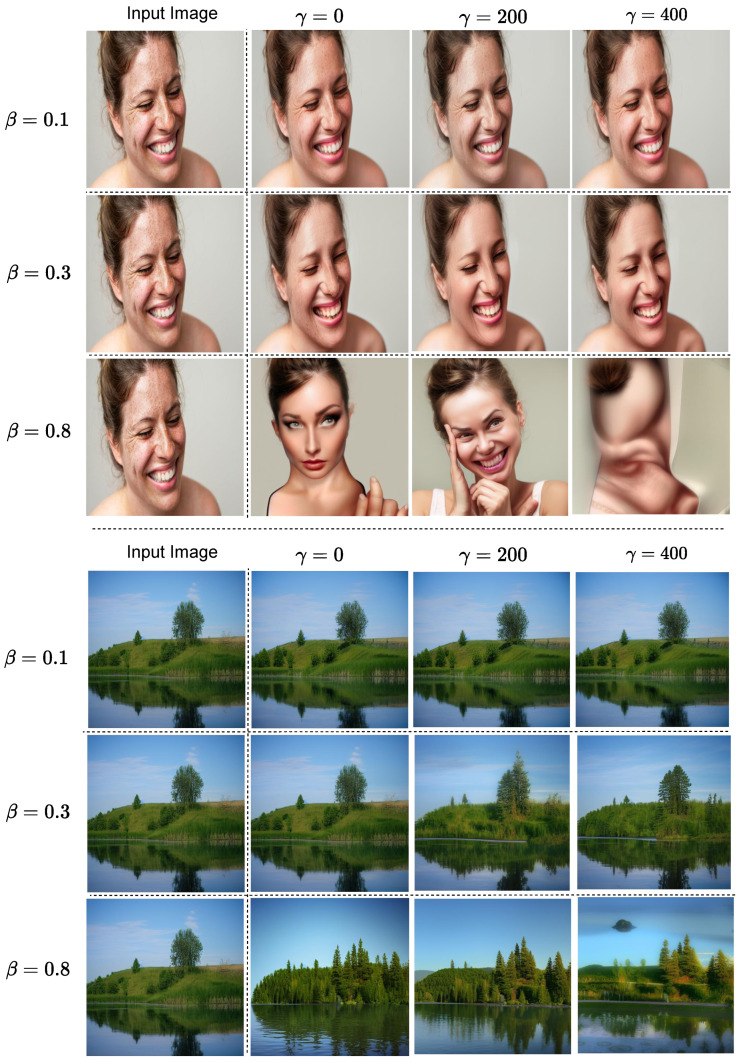
Robustness analysis under coupled concept strength γ and noise level β settings for the “perfect skin” and “peaceful lake” concepts. Rows correspond to different noise levels β, and columns correspond to different concept strengths γ. Low β preserves the input structure but limits semantic editability, moderate β provides a favorable balance between editing strength and fidelity, and high β combined with large γ may lead to identity drift, over-smoothing, or scene distortion.

As shown in Figure 8, low noise levels (β=0.1) preserve the input structure but limit the visible concept effect, even when γ is increased. Moderate noise levels (β=0.3) provide a more stable trade-off, allowing for clearer skin smoothing or a calmer lake appearance while preserving the main facial or scene structure. In contrast, high noise levels (β=0.8) weaken the structural constraint from the input image. When combined with large concept strengths (γ=200 or γ=400), this may cause identity drift, over-smoothing, or unrealistic scene distortion. These results show that stable semantic manipulation requires balancing the structure-preserving role of β with the semantic strength controlled by γ.

#### 4.5.2. Effect of Denoising-Stage-Dependent Concept Strength

We further evaluate whether the concept strength should remain fixed during denoising or vary across denoising stages. Instead of using a constant γ at all timesteps, we compare fixed-concept injection with timestep-dependent schedules, denoted as $\gamma_{t}$. The comparison is conducted for the “peaceful lake” concept using the same input image and the same image-to-image noise strength.

**Figure 9:**
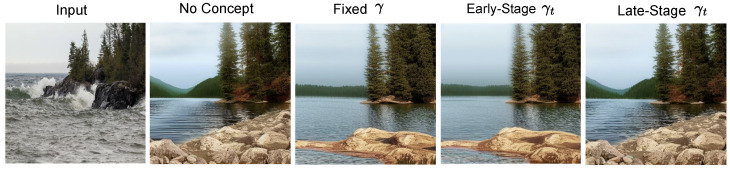
Effect of denoising-stage-dependent concept strength for the “peaceful lake” concept. The no-concept output provides the image-to-image baseline, while fixed and timestep-dependent concept injection introduce the learned concept vector during denoising. Early-stage injection produces stronger global atmospheric changes, whereas late-stage injection is more conservative and preserves more of the scene layout. All edited outputs are generated with β=0.8, and the maximum concept strength is set to γ=150.

As shown in Figure 9, the no-concept output provides the image-to-image baseline without concept-vector injection. Fixed-concept injection introduces the learned vector throughout the denoising process and produces a stable concept effect. Early-stage injection produces stronger global atmospheric changes because the concept vector affects the denoising trajectory while the main scene structure is still being formed. In contrast, late-stage injection is more conservative and mainly influences later visual refinement, preserving more of the scene layout. These results show that timestep-dependent concept strength is feasible, but fixed γ provides a simple and stable default setting for the main experiments.

#### 4.5.3. Ablation Study on Injection Location

To justify the choice of bottleneck-level injection, we compare four U-Net injection locations: down blocks, mid-block, up blocks, and all blocks. The ablation is conducted on the “perfect skin” concept using 67 real images, with the same learned concept vector, β=0.3, and γ=100. As shown in Table 5, mid-block injection achieves the best performance across all metrics, with an SSIM of 0.7023, an LPIPS of 0.2370, and a CLIP similarity of 0.2491. Down-block injection reduces perceptual quality and semantic alignment, while up-block and all-block injection cause severe degradation. This result supports the use of the U-Net bottleneck as a stable location for injecting a global concept vector.

**Figure 10:**
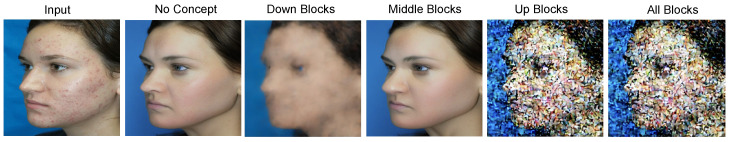
Qualitative comparison of different concept-vector injection locations for the “perfect skin” concept. Down-block injection weakens facial structure and concept control, while up-block and all-block injection cause severe artifacts and structural collapse. Mid-block injection provides the most stable result, preserving facial structure while enabling the learned concept effect.

Figure 10 provides a qualitative comparison of the different injection locations. Down-block injection weakens facial structure and concept control, while up-block and all-block injection produce severe artifacts and structural collapse. In contrast, mid-block injection preserves the main facial structure while enabling a stable concept effect, consistent with the quantitative results.

#### 4.5.4. Ablation Study on Multi-Prompt Pool

To evaluate the contribution of the proposed multi-prompt pool, we compare it with a single-prompt training setting. In the single-prompt setting, each concept is trained using one positive prompt and one neutral prompt. In the multi-prompt setting, the proposed positive and neutral prompt pools are used to provide broader semantic and appearance diversity during concept-vector learning.

As shown in Table 6, the multi-prompt pool improves CLIP similarity for both concepts, increasing from 0.2411 to 0.2491 for *perfect skin* and from 0.2678 to 0.2871 for *peaceful lake*. This indicates that using multiple positive and neutral prompts helps learn a concept vector that is more strongly aligned with the intended semantic direction. For *perfect skin*, the multi-prompt setting also improves SSIM and LPIPS, suggesting stronger concept alignment without reducing structural or perceptual consistency. For *peaceful lake*, the single-prompt setting obtains higher SSIM and lower LPIPS, indicating a more conservative reconstruction closer to the input image. However, the multi-prompt pool achieves higher CLIP similarity, suggesting a stronger semantic shift toward the intended peaceful atmosphere. Overall, the ablation supports the use of the multi-prompt pool for learning broader concept-level semantics, while also showing that subjective scene-level concepts may involve a stronger fidelity–semantics trade-off.

### 4.6. Parameter Efficiency and Computational Overhead

To substantiate the parameter-efficiency claim, we benchmark the proposed method against the Stable Diffusion image-to-image baseline under the same resolution and denoising settings. Since the pretrained VAE, text encoder, and U-Net remain frozen, the proposed method introduces only one learnable concept vector for each target concept. In our implementation, this vector has dimensionality 1280, resulting in only 1280 trainable parameters and approximately 0.0049 MB of additional fp32 storage.

Table 7 summarizes the parameter count, storage requirement, training time, and inference latency. Training the “perfect skin” concept vector requires 586 s. During inference, latency increases from 0.5858–0.6459 s/image for Stable Diffusion Img2Img to 0.6234–0.6821 s/image for the proposed method, corresponding to a small overhead of 6.43% for “perfect skin” and 5.62% for “peaceful lake”. Peak VRAM remains essentially unchanged, with 3.486 GB for Stable Diffusion Img2Img and 3.485 GB for the proposed method. These results show that the learned concept vector provides a lightweight adaptation mechanism with minimal storage and inference overhead.

## 5. Discussion

### 5.1. Analysis of Results

The experimental results show that the proposed concept-vector learning framework can improve semantic alignment while preserving the main structure of the input image. For the “perfect skin” concept, the method improves SSIM, LPIPS, and CLIP compared with the Stable Diffusion image-to-image baseline, indicating better structural preservation, perceptual consistency, and semantic alignment. For the “peaceful lake” concept, the proposed method achieves higher SSIM and CLIP similarity, while Stable Diffusion obtains lower LPIPS because it remains closer to the input image and performs a more conservative transformation. This confirms that subjective concept editing should be evaluated using multiple metrics rather than a single score. The qualitative results and human preference study further support the quantitative findings. The concept-injected outputs are preferred by human participants for both main concepts, indicating that the learned vectors capture perceptually meaningful changes. The additional portrait-level examples, including “melancholic portrait” and “dramatic portrait”, also suggest that the framework can extend beyond texture-level and scene-level edits to subjective visual attributes. However, the results also show that the strength of the edit depends on the interaction between the image-to-image noise strength β and the concept strength γ. Moderate settings provide the best balance between semantic modification and structural fidelity, while extreme settings can introduce visible artifacts.

### 5.2. Generalization Boundary for Geometric Concepts

**Figure 11:**
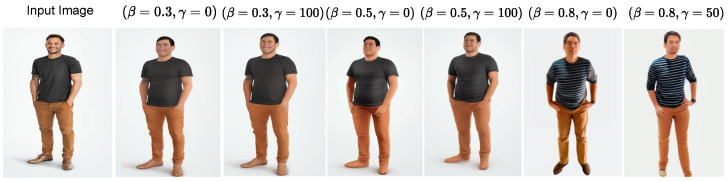
Generalization boundary analysis on the geometric concept “longer legs”. Unlike appearance-level concepts such as skin smoothness or scene atmosphere, this concept requires structured body deformation. At moderate noise levels, the input structure is largely preserved, but the intended leg-length change remains weak or inconsistent. Increasing β provides more generative freedom, but also introduces changes in identity, clothing, pose, and body structure. This result indicates that the proposed global bottleneck concept vector is more suitable for global appearance and atmosphere manipulation than precise geometry-aware editing.

To examine the boundary of the proposed framework, we evaluate a geometric concept, “longer legs”. Unlike appearance-level or atmosphere-level concepts, this task requires structured deformation of the human body. It therefore provides a challenging case for testing whether a single global bottleneck concept vector can support geometry-aware editing. As shown in Figure 11, the method has limited ability to produce precise geometric transformations. At moderate noise levels, the input structure is largely preserved, but the intended leg-length change remains weak or inconsistent. Increasing β provides more generative freedom, but it also introduces changes in identity, clothing, pose, and body structure. These results indicate that the proposed bottleneck concept vector is more suitable for global appearance, style, and atmosphere manipulation than precise geometry-aware editing.

### 5.3. Failure Cases

The robustness analysis in Figure 8 reveals representative failure cases under extreme parameter settings. When high noise strength (β=0.8) is combined with a large concept strength (γ=200 or γ=400), the denoising process may become unstable. For the “perfect skin” concept, this can lead to identity drift, excessive smoothing, or facial distortion. For the “peaceful lake” concept, it can produce unrealistic scene structures, distorted boundaries, or inconsistent textures. These failures mainly arise from feature over-perturbation. A larger β weakens the structural constraint from the input image, while an overly large γ can cause the concept vector to dominate the native denoising features. The injection-location ablation further supports this explanation, as up-block and all-block injection produce severe artifacts. Therefore, stable concept manipulation requires selecting moderate β and γ values and injecting the concept vector at a semantically meaningful but structurally stable representation level.

### 5.4. Limitations

Although the proposed framework provides a lightweight mechanism for concept-level manipulation, some limitations remain. First, the learned concept vector is injected into the U-Net bottleneck and therefore mainly acts as a global semantic perturbation. As a result, the method is more suitable for global appearance, style, and atmosphere manipulation than for precise region-specific editing, which may require masks, attention control, or region-aware conditioning. Second, the method has limited ability to perform precise geometric transformations. As shown in the “longer legs” boundary analysis, geometry-related concepts require changes in body proportions, pose, or spatial correspondence, which are difficult to represent using a single global bottleneck vector without reducing input fidelity. The method may also be less reliable for semantically conflicting or polysemous concepts, because a single additive concept vector can entangle incompatible visual attributes rather than separating them cleanly. Finally, the current empirical validation is conducted using Stable Diffusion 1.5, and each target concept requires learning a separate concept vector. Applying the method to other diffusion backbones, such as SDXL or domain-specific models, would require retraining the concept vector and reselecting γ and β. Future work will explore stronger spatial control, multi-concept composition, and adaptation to additional diffusion architectures.

## 6. Conclusions

This work presented a prompt-guided concept-vector learning framework for the controllable manipulation of abstract visual concepts in diffusion models. The method learns a compact concept vector in the U-Net bottleneck representation of Stable Diffusion while keeping the VAE, text encoder, and U-Net frozen. A positive–neutral multi-prompt strategy provides weak semantic guidance without requiring external human-annotated image pairs, segmentation masks, identity labels, or manually annotated editing targets. The learned vector is applied in an image-to-image editing pipeline through bottleneck-level feature injection, where γ controls concept strength and β balances structural preservation. Experiments on “perfect skin” and “peaceful lake” show improved semantic alignment and structural preservation compared with Stable Diffusion image-to-image baselines, while human preference results further support the perceptual effectiveness of the learned concept vectors. Additional results on “melancholic portrait” and “dramatic portrait” demonstrate applicability to subjective portrait-level concepts. Ablation and robustness analyses show that mid-block injection is the most stable location, multi-prompt learning improves semantic alignment, and moderate β–γ settings provide the best balance between editing strength and fidelity. Overall, the proposed framework provides a simple and parameter-efficient approach for global appearance and atmosphere manipulation. Future work will explore stronger spatial control, multi-concept composition, and adaptation to additional diffusion backbones.

## Figures and Tables

**Table 1:** Representative positive and neutral prompts used for multi-prompt synthetic dataset generation across the evaluated concepts.

Concept	Positive Prompts	Neutral Prompts
*Perfect skin*	A face with smooth and glowing skin; A portrait with healthy, flawless complexion	A face; A portrait of a person
*Peaceful lake*	A peaceful lake with calm still water; A serene lake with smooth reflections	A lake; A natural landscape with a lake
*Melancholic portrait*	A melancholic portrait with subdued emotional expression; A somber portrait with quiet sadness	A portrait of a person; A human face portrait
*Dramatic portrait*	A dramatic portrait with strong contrast lighting; A cinematic portrait with intense mood	A portrait of a person; A studio portrait

**Table 2:** Quantitative evaluation for the “perfect skin” concept on 67 real images.

Method	SSIM ↑	LPIPS ↓	CLIP ↑
Stable Diffusion	0.6636	0.2478	0.2460
Ours (w/o concept)	0.7011	**0.2314**	0.2481
Ours (with concept)	**0.7023**	0.2370	**0.2491**

Note: Bold values indicate the best performance. ↑ means higher is better, and ↓ means lower is better.

**Table 3:** Quantitative evaluation for the “peaceful lake” concept on 55 real images.

Method	SSIM ↑	LPIPS ↓	CLIP ↑
Stable Diffusion	0.4578	**0.4129**	0.2663
Ours (w/o concept)	0.4653	0.5086	0.2820
Ours (with concept)	**0.4854**	0.5021	**0.2871**

Note: Bold values indicate the best performance. ↑ means higher is better, and ↓ means lower is better.

**Table 4:** Method-level comparison with representative diffusion-based editing and concept-manipulation approaches.

Method	Supervision/ Training Data	Trainable Params.	Main Strength	Main Limitation
SDEdit/Stable Diffusion Img2Img [3,6]	No additional training; text prompt only	0	Simple image-to-image editing	Prompt-dependent control; weak for subjective concepts
Prompt-to-Prompt [13]	Text prompts and attention manipulation	0	Attention-based prompt editing	Sensitive to prompt alignment and attention maps
InstructPix2Pix [12]	Instruction-tuned external editing data	Pretrained editing model	Flexible instruction-based editing	May alter identity, background, or composition
Textual Inversion [25]	Concept-specific image set	Token embedding	Lightweight token-level learning	Limited stability for image-to-image control
Self-discovered latent directions [20]	Generated samples or latent-direction analysis	Method-dependent	Interpretable semantic directions	Target control can be indirect
Ours	Positive/neutral prompt pools without external human-annotated image pairs	1280 per concept	Explicit concept vector with continuous γ control	Global appearance control; less precise spatial editing

**Table 5:** Ablation study of concept-vector injection location for the “perfect skin” concept on 67 real images.

Injection Location	SSIM ↑	LPIPS ↓	CLIP ↑
Down blocks	0.6713	0.5188	0.2148
Mid-block	**0.7023**	**0.2370**	**0.2491**
Up blocks	0.1671	0.7972	0.1868
All blocks	0.1684	0.7952	0.1866

Note: Bold values indicate the best performance. ↑ means higher is better, and ↓ means lower is better.

**Table 6:** Ablation study comparing single-prompt training with the proposed multi-prompt pool.

Concept	Prompt Setting	SSIM ↑	LPIPS ↓	CLIP ↑
*Perfect skin*	Single prompt	0.6989	0.2671	0.2411
*Perfect skin*	Multi-prompt pool	**0.7023**	**0.2370**	**0.2491**
*Peaceful lake*	Single prompt	**0.5310**	**0.3264**	0.2678
*Peaceful lake*	Multi-prompt pool	0.4854	0.5021	**0.2871**

Note: Italics indicate target concept names; bold values indicate the best performance. ↑ means higher is better, and ↓ means lower is better.

**Table 7:** Parameter efficiency and inference overhead compared with the Stable Diffusion image-to-image baseline.

Method	Trainable Params.	Storage	Training	Latency/Image
Stable Diffusion Img2Img	0	0 MB	–	0.5858–0.6459 s
Ours	1280	0.0049 MB	586 s	0.6234–0.6821 s

## Data Availability

The code and generated datasets used in this study will be made publicly available upon acceptance. Additional data supporting the findings of this study are available from the corresponding author upon reasonable request.
